# Association of nighttime physical activity with all-cause and cardiovascular mortality: Results from the NHANES

**DOI:** 10.3389/fcvm.2022.918996

**Published:** 2022-08-05

**Authors:** Jiayi Yi, Lili Wang, Jiajun Guo, Ping Sun, Ping Shuai, Xiaoxiang Ma, Xiaojiao Zuo, Yuping Liu, Zhengwei Wan

**Affiliations:** ^1^Department of Cardiology, Fuwai Hospital, National Center for Cardiovascular Diseases, Chinese Academy of Medical Sciences and Peking Union Medical College, Beijing, China; ^2^Department of Cardiology, West China Hospital, Sichuan University, Chengdu, China; ^3^Department of Health Management & Institute of Health Management, Sichuan Provincial People's Hospital, University of Electronic Science and Technology of China, Chengdu, China; ^4^Chinese Academy of Sciences Sichuan Translational Medicine Research Hospital, Chengdu, China

**Keywords:** nighttime, National Health and Nutrition Examination Survey (NHANES), all-cause mortality, cardiovascular mortality, physical activities

## Abstract

**Background:**

Nighttime physical activity (PA) has significant effects on human health. Whether excessive nighttime PA is associated with adverse long-term prognosis remains unknown.

**Methods:**

Three thousand six hundred ninety adults from the US National Health and Nutrition Examination Survey (NHANES) 2003–2006 with accelerometer monitor recording PA data were included. Nighttime PA was quantified by the nighttime to all-day PA intensity ratio (NAPAIR). Participants with the NAPAIR above the population median (0.17) were defined as the nighttime active population (NAP), otherwise as the daytime active population. All-cause and cardiovascular disease mortality status was acquired from the US National Death Index from their interview and physical examination date through December 31, 2015.

**Results:**

Among 3690 adults (weighted mean age 48.1 years), 1781 (weighted proportion 48.8%) were females. One thousand eight hundred six (48.9%) were determined as the NAP. During the follow-up period of up to 13.1 years (median, 10.7 years), 639 deaths occurred (heart diseases, 114). Multivariable Cox proportional hazards model showed that the NAP was associated with higher risks of all-cause (hazard ratio [HR], 1.46; 95% confidence interval [CI], 1.22–1.75) and cardiovascular disease (HR, 1.58; 95% CI, 1.03–2.41) mortality compared with the daytime active population, and each 0.1 increase in the NAPAIR was associated with 15% increased all-cause mortality risks.

**Conclusion:**

In this nationally representative prospective cohort study of a sample of United States adults, excessive nighttime PA was associated with a higher risk of death from all causes and cardiovascular disease.

## Introduction

Physical activity (PA) positively affects blood pressure, blood glucose, and body weight (1). It has been proved that PA reduces the overall risk of incidence and mortality for cardiovascular disease, diabetes, ischemic stroke, and cancer (2–6). Given that PA is a safe and cost-effective health-promoting factor, 2020 WHO guidelines recommend moderate to vigorous PA across all age groups, including those living with chronic conditions or a disability (7, 8). PA guidelines provide recommendations for the types, intensity, volume, and duration of physical activity for both general and specific populations, while how to allocate all-day PA to daytime and nighttime was not mentioned.

Nighttime PA has significant effects on human circadian rhythm and the timing of PA should be considered. Previous studies have reported that PA at nighttime impacts neuroendocrine hormones, body temperature, and blood pressure. These changes further influence peripheral clocks, lead to the inconsistency between the main and the peripheral clocks, and disturb the circadian rhythm (9, 10). Abnormal circadian rhythm is associated with severe consequences, including metabolic disorders, sleep, psychiatric disorders, increased risk of heart attacks and cardiovascular events, and cancer (11–13). In addition, excessive PA at nighttime has adverse effects on sleep. Previous studies found that sleep was disturbed after nighttime exercise (14, 15). Furthermore, late-night activity may affect cardiac autonomy during the first sleeping hours (16). However, whether the excessive proportion of nighttime over all-day PA was associated with long-term survival remains unknown.

The National Health and Nutrition Examination Survey (NHANES) assesses the health and nutritional status of the civilian, non-institutionalized population in the US. During 2003–2006, the physical activity monitor (PAM) component was added to NHANES. The objective measurement of PA by accelerometry device is advantageous over self-report PA information for ameliorating respondents' perceptions bias. Furthermore, continuous 24-h PA records enable us to evaluate PA during daytime and nighttime. By NHANES, the US nationally representative sample, the present study aimed to explore the association between the proportion of nighttime over all-day PA with long-term survival.

## Methods

### Study design and population

The NHANES design and protocol have been described previously (17). The present study is a prospective cohort study that used a population of individuals from the 2003–2004 and 2005–2006 NHANES cycles (*n* = 20470) with physical activity monitor (PAM) data. Participants aged <20 years (n = 10450), with <4 valid PAM wearing days (*n* = 6325), or without mortality information (*n* = 5) were excluded, leaving a total of 3,690 participants for the present analysis (Supplementary Figure S1). This study was conducted and reported following the Strengthening the Reporting of Observational Studies in Epidemiology (STROBE) statement.

### Physical activity measures and definition

Physical activity was objectively assessed with an ActiGraph Model 7,164 accelerometer (ActiGraph, Ft. Walton Beach, FL). The uniaxial monitor measures and records vertical acceleration as counts for successive 1**-**min intervals beginning at 12:01 a.m. the day after the health examination, and all data were recorded in a 24-hour clock. Details of the accelerometer monitor protocol have been described previously (18, 19). In brief, participants were required to wear the hip**-**worn monitor during all waking hours for seven consecutive days. The nighttime was defined as 8:00 p.m. to 5:59 a.m. and the daytime as 6:00 a.m. to 7:59 PM. Monitor non-wear time was defined as intervals of at least 60 consecutive minutes of zero counts, and the definition was consistent with previous studies (19, 20). Monitor wearing time was estimated by subtracting non-wearing time from the total time for the day. A valid PAM wearing day was defined as a day with over 10 h wearing time during the day and 2 h wearing time during the nighttime. Nighttime physical activity was quantified by the nighttime to all-day physical activity intensity ratio (NAPAIR). The NAPAIR was defined as nighttime total PA intensity counts divided by all-day total PA intensity counts over all valid PAM wearing days. The NAPAIR ranges from 0 to 1, and the value indicates the proportion of participants' nighttime PA intensity to the all-day PA intensity. We used the median of the study population NAPAIR (0.17) as the cut-off point and divided the population into two groups. Participants with the NAPAIR ≥ 0.17 were considered to have excessive nighttime PA and were defined as the night-time active population (NAP). Participants with NAPAIR < 0.17 were defined as the daytime active population (DAP).

### Mortality outcome ascertainment

NCHS provided NHANES Public-Use Linked Mortality File, and it was linked to the National Death Index, an NCHS centralized database of all deaths in the United States, through December 31, 2015 (21). The underlying cause of death of participants was recorded by the definition of the International Statistical Classification of Diseases and Related Health Problems, Tenth Revision (ICD-10). Cardiovascular disease (CVD) mortality is death from heart disease (codes I00–I09, I11, I13, and I20–I51) (22). Mortality due to causes other than CVD was considered the competing risk event. The duration of follow-up was defined as the interval in months from the interview date to the date of death or through December 31, 2015, for participants without event.

### Covariates assessment

Sociodemographic characteristics were collected by questionnaires, including sex, age, race/ethnicity, educational attainment, marital status, and family poverty income ratio (total family income divided by the poverty threshold). Participants' weight and height were measured during the physical examination, and body mass index (BMI) was calculated as weight in kilograms divided by height in meters squared. Self-reported smoking status was classified into 3 groups following the NCHS definition: current smoker, former smoker, and never smoker (23). Health status was self-reported by participants and was categorized into three groups (excellent or good, fair, and poor). Hypertension and hypercholesterolemia were both determined either self-reported by participants who had been diagnosed by a health professional or by the NHANES objective measurements (Systolic blood pressure ≥ 140 mmHg or diastolic blood pressure ≥ 90 mmHg for hypertension; cholesterol ≥ 6.2 mmol/L for hypercholesterolemia). Diabetes was self-reported by participants who had been diagnosed by a health professional or determined by a prescription history for medications used to treat the condition (24). Overall average physical activity intensity (OAPAI) was calculated by summing all 1-min intervals PA intensity counts and dividing by the total number of minutes over all valid PAM wearing days.

### Statistical analysis

All analyses were conducted following the NHANES analytic guidelines. Sample weights, strata, and primary sampling units accounted for the unequal probability of selection, oversampling of specific subpopulations, and nonresponse adjustments were used to generate nationally representative estimates. Baseline characteristics were compared using the chi-square test for categorical variables. Survival curves were plotted with the Kaplan–Meier method. Cox proportional hazards regression models were applied to estimate hazard ratios (HRs) and 95% CIs for the associations between the NAPAIR and risk of all-cause and cardiovascular mortality. The proportional hazards assumption was examined by conducting the Schoenfeld test and creating a product term of follow-up time and NAPAIR group. To discriminate CVD-related deaths from non-CVD-related deaths, we also performed a competing risk analysis to evaluate the risk of CVD-related death using Fine-Gray proportional hazard models. Stratified and interaction analyses were performed to determine whether the association differed by age, sex, obesity and health status, and chronic comorbidities (hypertension, hypercholesterolemia, or diabetes). Finally, we conducted sensitivity analyses by excluding deaths that occurred during the first 2-year follow-up to reduce the possibility of reverse causation (25). We also conducted sensitivity analyses by changing the definition of nighttime to 7:00 p.m. to 6:59 a.m. and the daytime to 7:00 a.m. to 6:59 PM. Statistical tests were 2-sided, and statistical significance was set at *P* < 0.05. All statistical analyses were conducted using R software, version 4.0.5 (R Core Team, Vienna, Austria), and Python software, version 3.8.8 (G. van Rossum). Data analyses were conducted in February 2022.

## Results

### Baseline characteristics

This study included 3690 adults aged 20 years or older (weighted population, 85,953,737; weighted mean [SE] age, 48.1 [0.4] years; 1781 (weighted proportion [WP], 48.8% female). In the study cohort, 585 (WP, 5.7%) were Mexican American, 97 (WP, 3.0%) were other Hispanic, 2053 (WP, 75.1%) were non-Hispanic White, 782 (WP, 10.4%) were non-Hispanic Black, and 173 (WP, 5.8%) were individuals of “other” race or ethnicity (Table 1). One thousand eight hundred six (48.9%) participants were determined as the NAP and 1884 (51.1%) were classified as the DAP. More than half of the participants in the NAP group were aged 20 to 45 years (WP, 50.2%). The weighted proportion of participants over 65 years in the DAP group was higher than the NAP group (23.7 vs. 12.7%). The prevalence of moderate to severe obesity (BMI ≥ 30), current smoking, and diabetes was higher in the NAP group compared with the DAP group (BMI ≥ 30: WP 32.7 vs. 28.2%; current smoker: WP 24.8 vs. 18.0%; diabetes: WP 8.3 vs. 7.4%) while the prevalence of hypertension (40.8 vs. 33.8%) and hypercholesterolemia (36.7 vs. 29.7%) were higher in the DAP group (Table 1).

**Table 1:** Sample size and characteristics of the study population, according to the NAPAIR Levels^a^.

**Characteristic**	**No. of participants by the NAPAIR Levels (weighted %)^a^**			***P*** **value^b^**
	**Total population**	**DAP** **(NAPAIR 0~0.17)**	**NAP** **(NAPAIR 0.17~1)**	
	**(*N* = 3,690)**	**(*N* = 1,884)**	**(*N* = 1,806)**	
**Sex**				<0.01
Male	1,909 (51.2)	1,029 (52.9)	880 (49.3)	
Female	1,781 (48.8)	855 (47.1)	926 (50.7)	
**Age group, y**				<0.01
20–44	1,423 (43.0)	581 (36.3)	842 (50.2)	
45–64	1,198 (38.6)	615 (40.0)	583 (37.1)	
≥65	1,069 (18.4)	688 (23.7)	381 (12.7)	
**Race/ethnicity**				<0.01
Mexican American	585 (5.7)	317 (5.3)	268 (6.1)	
Other Hispanic	97 (3.0)	46 (2.8)	51 (3.2)	
Non-Hispanic White	2,053 (75.1)	1,170 (80.3)	883 (69.6)	
Non-Hispanic Black	782 (10.4)	278 (7.1)	504 (13.9)	
Other Race	173 (5.8)	73 (4.5)	100 (7.2)	
**Education attainment**				0.55
High school or below	1,716 (37.7)	886 (37.7)	830 (37.6)	
College or above	1,971 (62.3)	997 (62.3)	974 (62.3)	
**Marital status**				<0.01
Married or living with a partner	2,296 (65.7)	1,258 (69.9)	1,038 (61.3)	
Single	1,393 (34.2)	625 (30.0)	768 (38.7)	
**FPIR**				<0.01
<1.3	810 (14.9)	371 (12.2)	439 (17.8)	
1.3 to <3.5	1,414 (36.1)	726 (35.7)	688 (36.6)	
≥3.5	1,314 (45.6)	713 (49.0)	601 (41.9)	
**BMI group**				<0.01
<25	1,172 (34.0)	613 (33.8)	559 (34.2)	
25 to <30	1,330 (35.0)	728 (37.6)	602 (32.3)	
≥30	1,161 (30.4)	535 (28.2)	626 (32.7)	
**Health status**				<0.01
Excellent or good	2,806 (81.4)	1,510 (85.5)	1,296 (77.1)	
Fair	571 (11.3)	247 (9.1)	324 (13.6)	
Poor	105 (2.2)	41 (1.7)	64 (2.8)	
**Smoker**				<0.01
Current	728 (21.3)	316 (18.0)	412 (24.8)	
Former	1,043 (27.2)	614 (30.5)	429 (23.6)	
Never	1,918 (51.5)	954 (51.5)	964 (51.6)	
**Diabetes**				0.42
Yes	392 (7.8)	188 (7.4)	204 (8.3)	
No	3,231 (90.7)	1,663 (91.3)	1,568 (90.0)	
Borderline	64 (1.4)	33 (1.4)	31 (1.5)	
**Hypertension**				<0.01
Yes	1,595 (37.4)	879 (40.8)	716 (33.8)	
No	2,095 (62.6)	1,005 (59.2)	1,090 (66.2)	
**Hypercholesterolemia**				<0.01
Yes	1,287 (33.3)	738 (36.7)	549 (29.7)	
No	2,403 (66.7)	1,146 (63.3)	1,257 (70.3)	

*NAPAIR, Nighttime to all-day physical activity intensity ratio; NAP, nighttime active population; DAP, daytime active population; BMI, body mass index; FPIR, family poverty income ratio*.

a *Weighted to be nationally representative. The weighted percentage may not sum to 100% because of missing data*.

b *P values of chi-square test were calculated based on the unweighted values*.

### PA intensity and wearing time recorded by PAM

**Figure 1:**
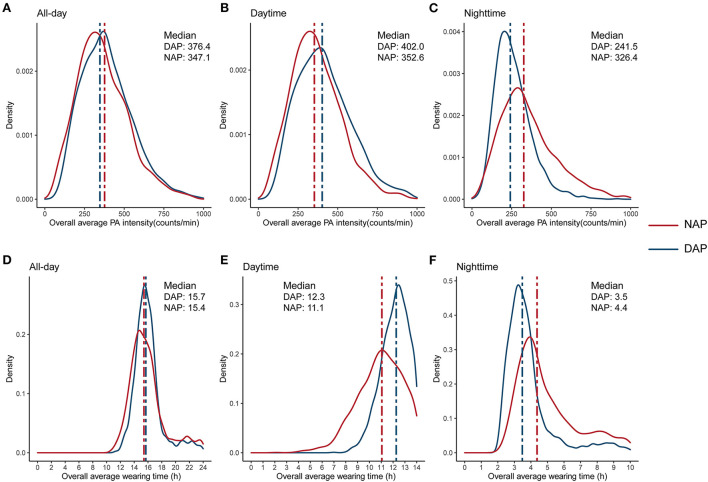
Distribution of overall average physical activity intensity **(A–C)**, and overall average wearing time **(D–F)**, by the NAPAIR groups. NAPAIR, Nighttime to all-day physical activity intensity ratio; NAP, nighttime active population; DAP, daytime active population.

The average valid PAM wearing days was 5.52 in the total population, with the median overall average wearing time of 15.58 hours. The median of overall all-day average PA intensity was 362.54 counts/min (Table 2). The density plots demonstrated that both groups have similar overall all-day average wearing time and overall all-day average PA intensity. However, the NAP group has a 1.2 h longer wearing time during the nighttime, and this gap was mostly compensated during the daytime (1.1 h). Similar patterns were also demonstrated in overall average PA intensity in nighttime and daytime (Figure 1).

**Table 2:** Physical activity monitors recording information of the study population, according to the NAPAIR Levels.

**PAM Recording Information**	**Total population**	**DAP (NAPAIR <0.17)**	**NAP (NAPAIR≥0.17)**
	**(*N* = 3,690)**	**(*N* = 1,884)**	**(*N* = 1,806)**
Average valid wearing days, mean (SD)	5.52 (1.12)	5.43 (1.12)	5.62 (1.12)
**Overall average PA intensity per day, median (IQR), counts/min**			
All-day	362.54 (217.9)	376.38 (214.53)	347.11 (217.53)
Daytime	373.74 (229.45)	402.04 (241.3)	352.56 (211.55)
Nighttime	277.77 (187.11)	241.47 (142.34)	326.37 (229.9)
**Overall average wearing time per day, median (IQR), hours**			
All-day	15.58 (2.22)	15.69 (1.93)	15.42 (2.57)
Daytime	11.78 (2.2)	12.27 (1.66)	11.05 (2.69)
Nighttime	3.88 (1.77)	3.48 (1.13)	4.37 (2.19)

*PAM, physical activity monitor; SD, standard deviation; IQR, interquartile range; DAP, daytime active population; NAP, nighttime active population; NAPAIR, Nighttime to all-day physical activity intensity ratio*.

### Survival analysis

During the 37,457 person-years follow-up (median follow-up was 10.7 years; maximum follow-up was 13.1 years), 639 deaths occurred, including 114 deaths from CVD. The NAP group participants were at a higher risk of death. In the age-adjusted model (model 1), participants in the NAP group had a 53% higher risk of all-cause mortality (hazard ratio (HR): 1.53, 95% confidence interval (CI): 1.31–1.80) and 77% higher risk of CVD mortality risk (HR: 1.77, 95% CI: 1.22–2.56). Each 0.1 increase in the NAPAIR was associated with 24 and 21% increased risks of death from all-cause and CVD, respectively. In model 2, we further adjusted sociodemographic characteristics, including sex, race/ethnicity, education attainment, marital status, and family poverty income ratio (FPIR). HRs for all-cause mortality and CVD mortality among the NAP group compared with the DAP group were 1.60 (95% CI: 1.35–1.89) and 1.87 (95% CI: 1.27–2.76), and each 0.1 increase in the NAPAIR was associated with 23 and 21% increased all-cause and CVD mortality risks. After additionally adjusted health behaviors and chronic conditions including BMI, health status, smoking status, diabetes, hypercholesterolemia, hypercholesterolemia, and overall average PA intensity in model 3, the NAP group remained a significant independent risk factor for all-cause mortality (HR: 1.46, 95% CI: 1.22–1.75) and CVD mortality (HR: 1.58, 95% CI: 1.03–2.41), and each 0.1 increase in the NAPAIR was associated with 15% increased all-cause mortality risks (Table 3). In competing risk analysis, the NAP group as a risk factor for CVD mortality was only significant in model 1 (HR: 1.58, 95% CI: 1.08–2.29) and model 2 (HR: 1.61, 95%CI: 1.09–2.34) while the NAPAIR was not significant in all three models (Supplementary Table S1).

**Table 3:** Association of the NAPAIR with all-cause and cardiovascular disease mortality in US adults.

**Mortality outcome**	**Death/No**.	**Weighted death (%)**	**Hazard ratio (95% CI)**		
			**Model 1^a^**	**Model 2^b^**	**Model 3^c^**
**All causes**					
**NAPAIR**					
<0.17	347/1,884	4,912,315 (11.1)	1[Reference]	1[Reference]	1[Reference]
≥0.17	292/1,806	4,184,788 (10.1)	1.53 (1.31–1.80)	1.60 (1.35–1.89)	1.46 (1.22–1.75)
Per 0.1 increase	NA	NA	1.24 (1.15–1.33)	1.23 (1.14–1.33)	1.15 (1.06–1.26)
**Cardiovascular death**					
**NAPAIR**					
<0.17	58/1,884	849,221 (1.9)	1[Reference]	1[Reference]	1[Reference]
≥0.17	56/1,806	721,872 (1.7)	1.77 (1.22–2.56)	1.87 (1.27–2.76)	1.58 (1.03–2.41)
Per 0.1 increase	NA	NA	1.21 (1.01–1.45)	1.21 (1.01–1.44)	1.09 (0.88–1.34)

*NAPAIR, Nighttime to all-day physical activity intensity ratio*.

a *Adjusted for age (as a continuous variable)*.

b *Additionally adjusted for sex, race/ethnicity, education attainment, marital status, and family poverty income ratio (as a continuous variable)*.

c *Additionally adjusted for body mass index (as a continuous variable), health status, smoking status, diabetes, hypercholesterolemia, hypercholesterolemia, and overall average physical activity intensity (as a continuous variable)*.

**Figure 2:**
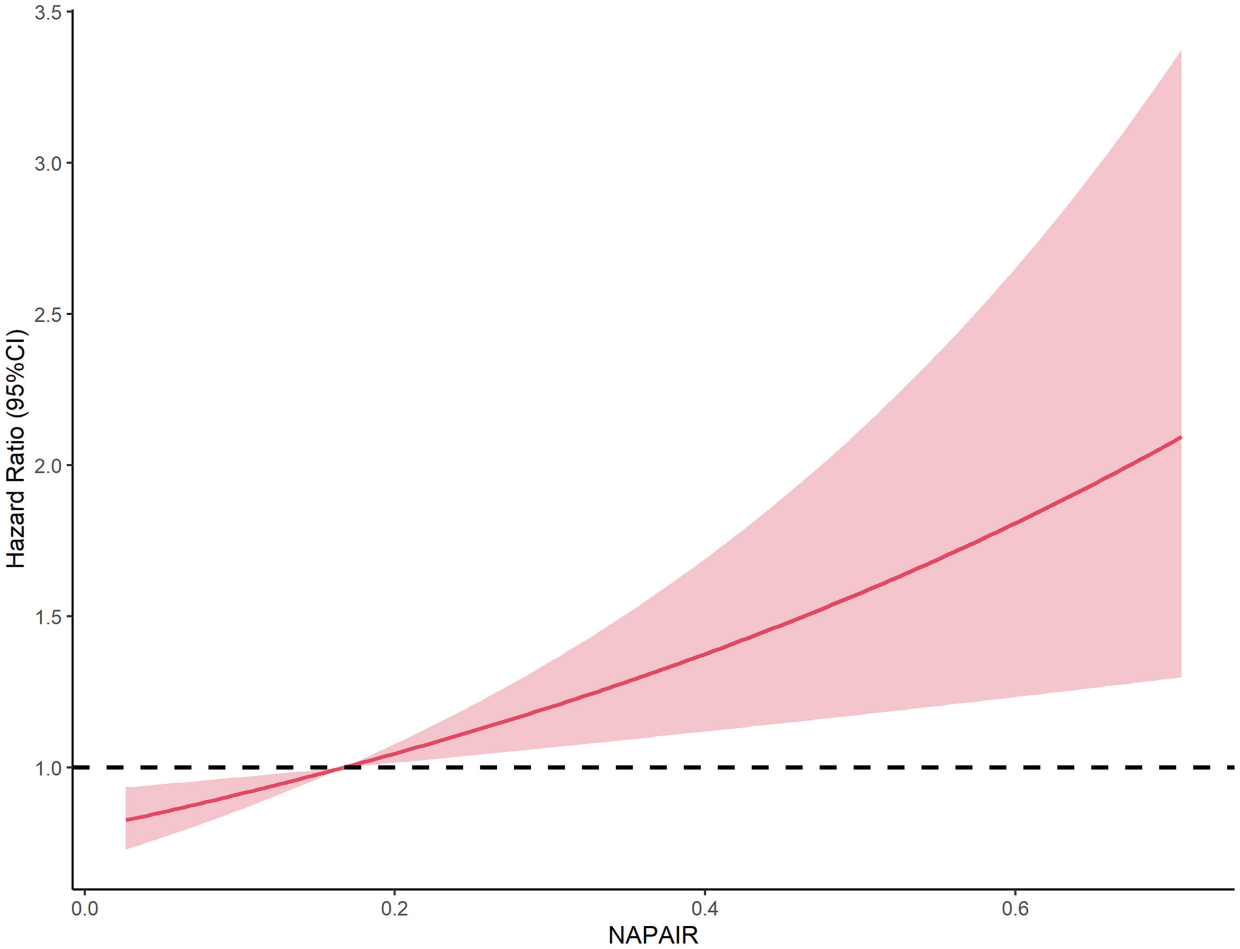
Dose-response association between the NAPAIR and all-cause mortality in US adults. NAPAIR, Nighttime to all-day physical activity intensity ratio.

We further investigated the dose-response association, stratified, interaction, and sensitivity analyses between NAPAIR and all-cause mortality of model 3. A J-shaped relationship was observed in dose-response analysis, indicating that mortality risk substantially increased when NAPAIR was higher than 0.17 (with estimated HR = 1) (Figure 2). Stratified analyses suggest that the observed associations of the NAPAIR with all-cause mortality did not significantly differ by age, sex, obesity, health status, and chronic comorbidities, nor a significant interaction effect was observed (Supplementary Table S2). All results remained similar in sensitivity analyses, excluding deaths that occurred during the first 2-year follow-up (Supplementary Tables S3–S5). The Schoenfeld test and product term of follow-up time and the NAPAIR group were applied in the three models described above and with all tests *p* ≥ 0.05, indicating no apparent violation of the proportional hazards assumption.

**Figure 3:**
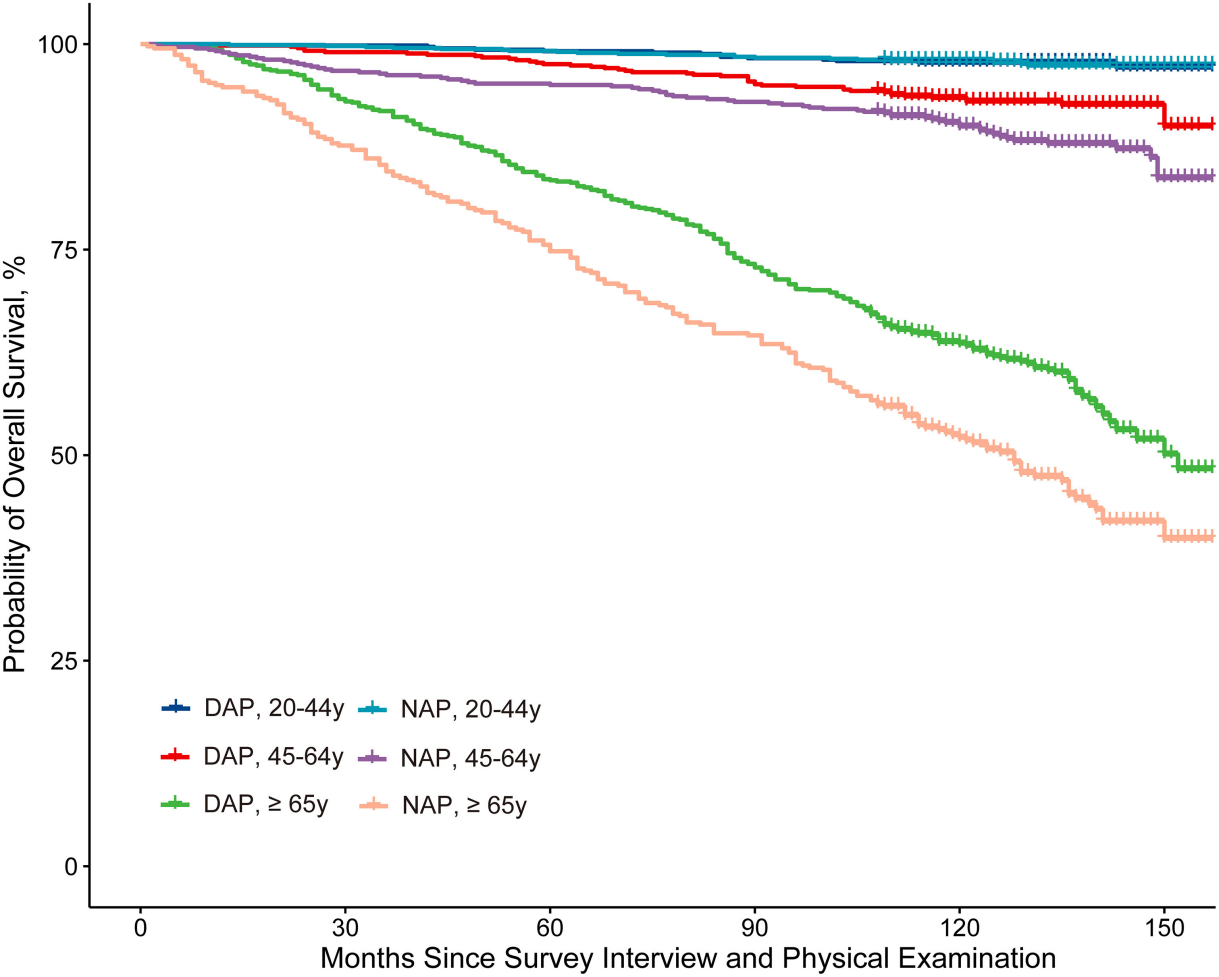
Kaplan-Meier curve analysis of all-cause mortality stratified by the NAPAIR and age group. NAPAIR, Nighttime to all-day physical activity intensity ratio; NAP, nighttime active population; DAP, daytime active population.

Kaplan–Meier curves for survival showed that the NAP group had a worse prognosis than the DAP group in general. The DAP had a higher survival rate among participants aged 45 years or older than the NAP. While among younger adults (aged 20–45 years), the difference in survival rates between the two groups was not apparent (Figure 3).

## Discussion

In this national representative prospective cohort of US adults, we determined an increased risk of all-cause and CVD mortality in the NAP. This association remained significant after adjustment for confounders. In addition, the results suggest that younger adults were more likely to be the NAP and have a higher prevalence of poor socioeconomic status, obesity, current smoking and diabetes, and longer nighttime activity duration.

To our knowledge, this is the first study to investigate the associations of nighttime PA intensity with long-term mortality outcomes. Previous studies have shown some implications of this finding. Excessive night PA has various effects on the body; sleep is most apparent. The association between night PA and sleep was deeply researched. In Cauter's study, the author demonstrates that nighttime physical exercise can delay the onset of nocturnal melatonin secretion, a robust endocrine marker of the circadian phase (15). Youngstedt's study also found that profound sleep was disturbed after nighttime exercise (14). Based on these findings, experts have recommended not exercising at night as part of good sleep hygiene and suggest that one avoid exercising early as 4–6 h before bedtime (26, 27). Some published research obtained different results, they demonstrated that evening moderate or vigorous exercises were not associated with worse sleep, and evening exercise did not disrupt sleep (28, 29). In addition, research from Rusko et al. indicates that vigorous late-night exercise may affect the cardiac autonomy of the heart during the first sleeping hours (16). With adverse effects of excessive night PA on sleep and cardiac, it is reasonable to hypothesize that the population with excessive night PA is at increased mortality risk, while the direct evidence is lacking.

In stratified and interaction analysis, we found that the HR of the NAPAIR was marginal significantly higher (*p* = 0.06) in the poor health status group than in fair and better groups. This finding suggests that the dose-response effect of nighttime PA was not consistent in different health statuses people and nighttime PA could induce a higher risk in those who had worse health status. However, we also noticed that this effect might be the result of a reverse causation relationship. Patients had worse health status might be unable to conduct excessive PA in the daytime which “passively” caused higher NAPAIR. And the higher all-cause mortality might also be caused by their unmeasured comorbidities. Therefore, this result should be cautiously interpreted and further studies to elucidate this relationship were needed.

The present study demonstrated that the NAP was at increased risk of all-cause and CVD mortality. Possible reasons for these association are as follows: (1) Excessive nighttime PA disturb sleep rhythm, and it is proved that poor sleep was related to increased CVD and all-cause mortality risk (30); (2) Excessive nighttime PA affected glands activity and hormones production and the thyroid is one of the most affected glands (31). The abnormalities in thyroid hormones concentrations have profound effects on the cardiometabolic and hemodynamic systems (32), and it was reported to be associated with increased cardiovascular risk, especially risk of atrial fibrillation (33, 34) which could also increase the mortality risk; (3) Excessive nighttime PA have effects on cardiac autonomic of heart (16), which may have an impact on long-term survival.

Our findings may have some public health implications. In the NAP group of the study cohort, more than half of them aged <45, and the prevalence of moderate-to-severe obesity, smoking, and diabetes was higher than the DAP group. Nowadays, many young office workers tend to be sedentary during working hours and prefer to exercise off-duty time at night, which could incur a higher NAPAIR. According to the result of the present study, they might be exposed to increased mortality risk. However, NHANES PA measurement was not specified in the exercise. Future studies are needed to elucidate whether people should avoid exercise at night. In addition, the results of PAM recording information showed that the NAP and the DAP have comparable valid wearing duration throughout the day while the NAP has valid PAM wearing time 1.2 h shorter during the daytime and 1.1 h longer at night. According to the PAM protocol, participants were required to wear the PAM during all waking hours; we can infer that the NAP delayed bedtimes and wake-up times. Even if the delay of bedtimes were compensated in the daytime, the NAP still has increased mortality risk. The result may indicate the negative impacts of delayed bedtimes and wake-up times.

Another interesting finding of the study is that all-cause mortality risk substantially increased if NAPAIR exceeds 0.17. In the general population, it is reasonable to estimate the waking time at night as 4 h (from 8 p.m. to 24 p.m.), accounting for 22% of the total waking time of the whole day (18 h in total from 6 a.m. to 24 p.m.). The NAPAIR should be 0.22 if a person's activities are uniformly distributed throughout the day. With NAPAIR ≥ 0.17, the decrease rate of night PA intensity will be <23% compared with daytime PA intensity [(0.22–0.17) ÷ 0.22 = 23%]. Similar circadian rhythms were also observed in blood pressure (BP). The normal BP circadian rhythm was defined as the decreased rate of nighttime BP compared with daytime BP within 10–20%. The abnormal circadian rhythm of BP is an independent predictor of target organ damage, cardiovascular events, stroke, and death (35–38). Results in this study prove that PA-induced abnormal circadian rhythm was also associated with poor outcomes and emphasize maintaining the normal circadian rhythm.

### Strengths and limitations

The strength of this study was using a nationally representative sample of US adults, which allows the findings to be generalized to a broader population. Additionally, the PAM device's sensitivity and reliable measurement properties enable the objective estimation of monitor wearing and daytime and nighttime PA intensity by scanning the minute-by-minute data. Several limitations should also be considered. Firstly, the PAM device only records uniaxial movement; activity information recorded for persons who use stationary bikes, elliptical trainers, or equipment that primarily involved upper-body training may not be recorded accurately. Therefore, our findings may not generalize to those who prefer these exercises. Secondly, the PA data were assessed at baseline, which may not reflect behavioral changes during the follow-up period. Studies employing repeated measures are needed to evaluate the longitudinal effects of PA day-night patterns on mortality. Finally, although we found an interaction between health status and the nighttime PA, the participant's general health status was self-reported, and NHANES did not provide an objective evaluation. In addition, this effect might be the result of an inverse causal relationship. However, after excluding deaths occurring during the first 2–year follow–up period, our results remain consistent, which reduces the possibility of reverse causation deduction.

## Conclusion

In this prospective cohort study of a nationally representative sample of US adults, the higher nighttime to all-day PA intensity ratio was associated with increased all-cause and CVD mortality risks. This study has provided new evidence that day-night PA patterns should be considered in future observational, epidemiologic, and intervention studies.

## Data availability statement

The National Health and Nutrition Examination Survey dataset are publicly available at the National Center for Health Statistics of the Center for Disease Control and Prevention (https://www.cdc.gov/nchs/nhanes/index.htm).

## Ethics statement

Ethical review and approval was not required for the study on human participants in accordance with the local legislation and institutional requirements. Written informed consent for participation was not required for this study in accordance with the national legislation and the institutional requirements.

## Author contributions

Material preparation, data collection, and analysis were performed by JY. All authors contributed to the study's conception and design, wrote the first draft of the manuscript, commented on previous versions of the manuscript, read, and approved the final manuscript.

## Funding

The study was supported by the National Science Foundation for Young Scholars of Sichuan Provincial People's Hospital (Grant No. 2022QN18), the Scientific Research Project of Sichuan Provincial Health Commission (No. 20PJ107), and the Scientific Research Project of China Health Promotion Foundation (2019).

## Conflict of interest

The authors declare that the research was conducted in the absence of any commercial or financial relationships that could be construed as a potential conflict of interest.

## Publisher's note

All claims expressed in this article are solely those of the authors and do not necessarily represent those of their affiliated organizations, or those of the publisher, the editors and the reviewers. Any product that may be evaluated in this article, or claim that may be made by its manufacturer, is not guaranteed or endorsed by the publisher.
