# Supplementary material for: Association of nighttime physical activity with all-cause and cardiovascular mortality: Results from the NHANES
Source: Front Cardiovasc Med. 2022 Aug 5;9:918996. doi: 10.3389/fcvm.2022.918996 (PMC9388927; doi:10.3389/fcvm.2022.918996)
Supplement: Supplementary file 1 [file Data_Sheet_1.docx]

**SUPPLEMENTARY**

**Figure S1**. Flow chart of the screening process for the selection of study population.


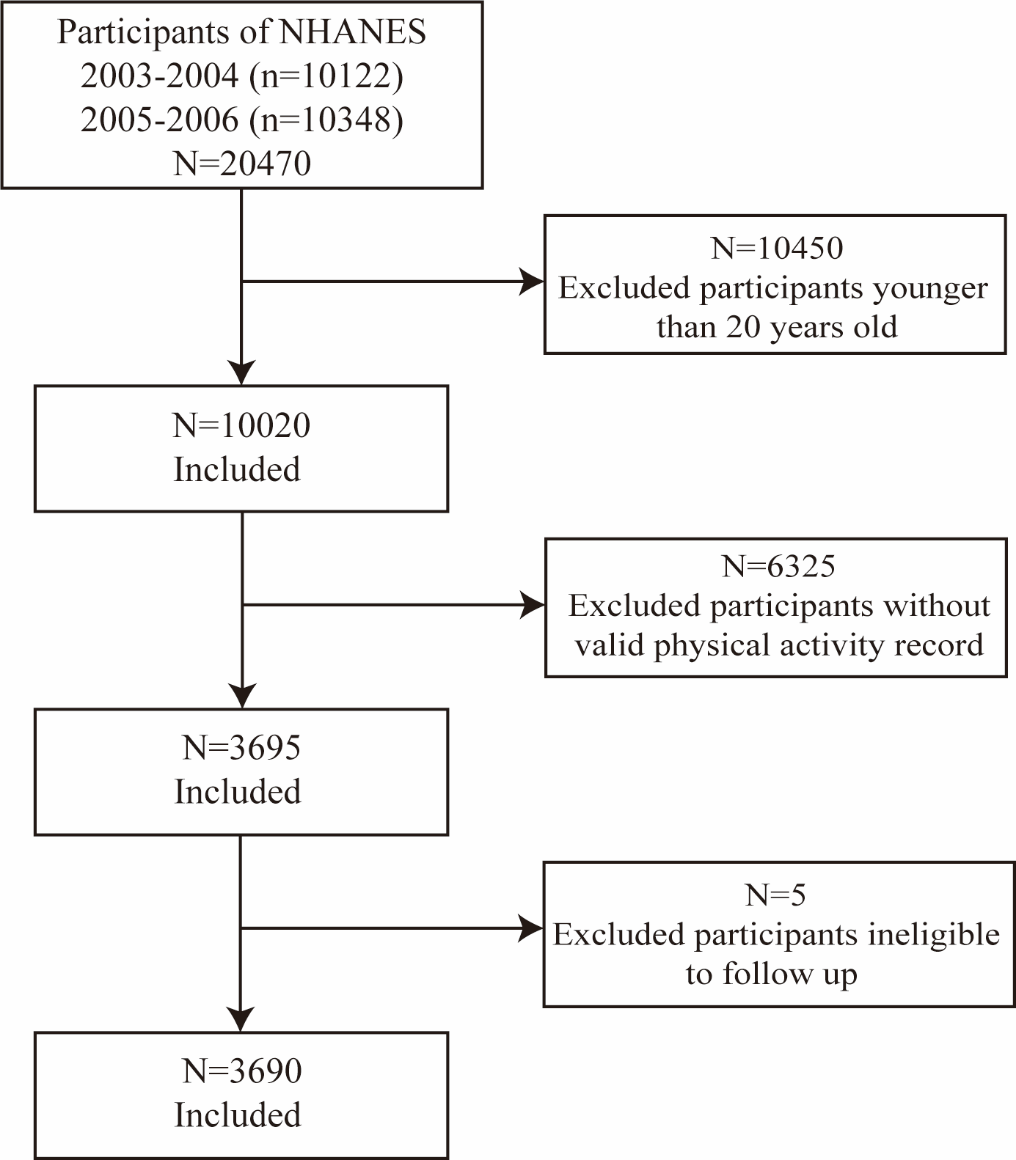


**Table S1.** Competing risk analysis of the association of the NAPAIR with cardiovascular and non-cardiovascular mortality in US adults.

| **Mortality outcome** | **Hazard ratio (95% CI)** | | |
| --- | --- | --- | --- |
|  | **Model 1^a^** | **Model 2^b^** | **Model 3^c^** |
| **Cardiovascular death** |  |  |  |
| **NAPAIR** |  |  |  |
| <0.17 | 1[Reference] | 1[Reference] | 1[Reference] |
| ≥0.17 | 1.58 (1.08–2.29) | 1.61 (1.09–2.34) | 1.39 (0.91–2.12) |
| Per 0.1 increase | 1.14 (0.97–1.34) | 1.11 (0.96–1.29) | 1.03 (0.85–1.25) |

Abbreviations: NAPAIR, Nighttime to all-day physical activity intensity ratio;

^a^ Adjusted for age (as a continuous variable).

^b^ Additionally adjusted for sex, race/ethnicity, education attainment, marital status, family poverty income ratio (as a continuous variable).

^c^ Additionally adjusted for body mass index (as a continuous variable), health status, smoking status, diabetes, hypercholesterolemia, hypercholesterolemia, and overall average physical activity intensity (as a continuous variable).

**Table S2.** Stratified and interaction analyses for the association of the NAPAIR with all-cause mortality of model 3

| **Variable** | **Hazard ratio (95% CI)** | | | **P-value for interaction** |
| --- | --- | --- | --- | --- |
|  | **DAP** | **NAP** | **Per 0.1 increase** |  |
| **Sex** |  |  |  |  |
| Male | 1[Reference] | 1.31 (1.04–1.66) | 1.08 (0.96–1.21) | 0.72 |
| Female | 1[Reference] | 1.59 (1.19–2.12) | 1.26 (1.08–1.46) |  |
| **Age group** |  |  |  |  |
| 20 to 44 | 1[Reference] | 0.98 (0.46–2.08) | 0.93 (0.70–1.23) | 0.66 |
| 45 to 64 | 1[Reference] | 1.86 (1.20–2.89) | 1.09 (0.91–1.29) |  |
| ≥65 | 1[Reference] | 1.41 (1.14–1.74) | 1.20 (1.07–1.34) |  |
| **Marital status** |  |  |  |  |
| Married or living with a partner | 1[Reference] | 1.43 (1.12–1.82) | 1.14 (1.01–1.29) | 0.35 |
| Single | 1[Reference] | 1.51 (1.15–1.97) | 1.17 (1.03–1.33) |  |
| **FPIR** |  |  |  |  |
| <1.3 | 1[Reference] | 1.52 (1.09–2.12) | 1.21 (1.03–1.41) | 0.40 |
| 1.3 to <3.5 | 1[Reference] | 1.62 (1.26–2.09) | 1.19 (1.06–1.34) |  |
| ≥3.5 | 1[Reference] | 1.19 (0.77–1.82) | 0.97 (0.77–1.23) |  |
| **BMI group** |  |  |  |  |
| <25 | 1[Reference] | 1.60 (1.13–2.27) | 1.11 (0.94–1.30) | 0.47 |
| 25 to <30 | 1[Reference] | 1.04 (0.78–1.40) | 1.03 (0.87–1.21) |  |
| ≥30 | 1[Reference] | 1.68 (1.20–2.37) | 1.23 (1.08–1.40) |  |
| **Health status** |  |  |  |  |
| Excellent or good | 1[Reference] | 1.48 (1.18–1.82) | 1.12 (1.00–1.25) | 0.06 |
| Fair | 1[Reference] | 1.15 (0.80–1.63) | 1.12 (0.96–1.32) |  |
| Poor | 1[Reference] | 3.49 (1.38–8.83) | 1.66 (1.15–2.40) |  |
| **Smoker** |  |  |  |  |
| Current | 1[Reference] | 1.21 (0.77–1.89) | 1.03 (0.84–1.25) | 0.85 |
| Former | 1[Reference] | 1.51 (1.15–1.98) | 1.22 (1.08–1.38) |  |
| Never | 1[Reference] | 1.33 (0.98–1.80) | 1.12 (0.95–1.31) |  |
| **Chronic** **comorbidities** |  |  |  |  |
| Yes | 1[Reference] | 1.45 (1.19–1.77) | 1.14 (1.03–1.27) | 0.61 |
| No | 1[Reference] | 1.43 (0.92–2.20) | 1.18 (0.98–1.41) |  |

Abbreviations: NAPAIR, Nighttime to all-day physical activity intensity ratio; DAP, daytime active population; NAP, nighttime active population; FPIR, family poverty income ratio; BMI, body mass index.

**Table S3.** Sensitive analysis for the association of the NAPAIR with all-cause mortality by excluding deaths that occurred during the first 2-year follow-up

| **All-causes Mortality** | **Death/No.** | **Weighted death (%)** | **Hazard ratio (95% CI)** | | |
| --- | --- | --- | --- | --- | --- |
|  |  |  | **Model 1^a^** | **Model 2^b^** | **Model 3^c^** |
| **NAPAIR** |  |  |  |  |  |
| < 0.17 (DAP) | 311/1848 | 4317078 (9.9) | 1[Reference] | 1[Reference] | 1[Reference] |
| ≥0.17 (NAP) | 239/1753 | 3473948 (8.5) | 1.41 (1.20–1.68) | 1.47 (1.23–1.77) | 1.37 (1.12–1.66) |
| Per 0.1 increase | NA | NA | 1.21 (1.11–1.31) | 1.21 (1.11–1.31) | 1.13 (1.03–1.25) |

Abbreviations: NAPAIR, Nighttime to all-day physical activity intensity ratio;

^a^ Adjusted for age (as a continuous variable).

^b^ Additionally adjusted for sex, race/ethnicity**,** education attainment, marital status, family poverty income ratio (as a continuous variable).

^c^ Additionally adjusted for body mass index (as a continuous variable), health status, smoking status, diabetes, hypercholesterolemia, hypertension, and overall average physical activity intensity (as a continuous variable).

**Supplement Results**

The following analysis results were based on the definition of nighttime as 7:00 PM to 6:59 AM:

**Table S4.** Sample Size and Characteristics of the Study Population, According to NAPAIR Levels.

| **Characteristic** | **No. of participants by NAPAIR Levels (weighted %)** ^a^ | | | **P value** |
| --- | --- | --- | --- | --- |
|  | **Total population** | **DAP**  **(NAPAIR 0~0.13)** | **NAP**  **(NAPAIR 0.13~1)** |  |
|  | （N=4724) | (N= 2300) | (N= 2424) |  |
| **Sex** |  |  |  |  |
| **Male** | 2431 (51.5) | 1291 (56.1) | 1140 (47.0) | <0.001 |
| **Female** | 2293 (48.5) | 1009 (43.9) | 1284 (53.0) |  |
| **Age group, y** |  |  |  |  |
| 20 to 44 | 1767 (37.4) | 733 (31.9) | 1034 (42.7) | <0.001 |
| 45 to 64 | 1548 (32.8) | 770 (33.5) | 778 (32.1) |  |
| ≥65 | 1409 (29.8) | 797 (34.7) | 612 (25.2) |  |
| **Race/ethnicity** |  |  |  | <0.001 |
| Mexican American | 872 (18.5) | 498 (21.7) | 374 (15.4) |  |
| Other Hispanic | 121 ( 2.6) | 55 ( 2.4) | 66 ( 2.7) |  |
| Non-Hispanic White | 2650 (56.1) | 1388 (60.3) | 1262 (52.1) |  |
| Non-Hispanic Black | 885 (18.7) | 297 (12.9) | 588 (24.3) |  |
| Other Race | 196 ( 4.1) | 62 ( 2.7) | 134 ( 5.5) |  |
| **Education attainment** |  |  |  | <0.001 |
| High school or below | 2285 (48.4) | 1191 (51.8) | 1094 (45.2) |  |
| College or above | 2436 (51.6) | 1109 (48.2) | 1327 (54.8) |  |
| **Marital status** |  |  |  | <0.001 |
| Married or living with a partner | 3080 (65.2) | 1627 (70.8) | 1453 (60.0) |  |
| Single | 1642 (34.8) | 672 (29.2) | 970 (40.0) |  |
| **FPIR** |  |  |  | 0.050 |
| ＜1.3 | 996 (22.0) | 472 (21.3) | 524 (22.7) |  |
| 1.3 to ＜3.5 | 1812 (40.0) | 863 (38.9) | 949 (41.1) |  |
| ≥3.5 | 1720 (38.0) | 882 (39.8) | 838 (36.3) |  |
| **BMI group** |  |  |  | <0.001 |
| ＜25 | 1464 (31.2) | 732 (32.0) | 732 (30.5) |  |
| 25 to ＜30 | 1729 (36.9) | 890 (38.9) | 839 (35.0) |  |
| ≥30 | 1497 (31.9) | 668 (29.2) | 829 (34.5) |  |
| **Health status** |  |  |  | 0.017 |
| Excellent or good | 3620 (81.3) | 1806 (82.6) | 1814 (80.0) |  |
| Fair | 712 (16.0) | 334 (15.3) | 378 (16.7) |  |
| Poor | 123 ( 2.8) | 47 ( 2.1) | 76 ( 3.4) |  |
| **Smoker** |  |  |  | <0.001 |
| Current | 875 (18.5) | 384 (16.7) | 491 (20.3) |  |
| Former | 1372 (29.0) | 737 (32.0) | 635 (26.2) |  |
| Never | 2476 (52.4) | 1179 (51.3) | 1297 (53.5) |  |
| **Diabetes** |  |  |  | 0.576 |
| Yes | 507 (10.7) | 236 (10.3) | 271 (11.2) |  |
| No | 4140 (87.7) | 2027 (88.1) | 2113 (87.3) |  |
| Borderline | 74 ( 1.6) | 37 ( 1.6) | 37 ( 1.5) |  |
| **Hypertension** |  |  |  | 0.002 |
| Yes | 2657 (56.2) | 1240 (53.9) | 1417 (58.5) |  |
| No | 2067 (43.8) | 1060 (46.1) | 1007 (41.5) |  |
| **Hypercholesterolemia** |  |  |  | <0.001 |
| Yes | 3021 (64.0) | 1389 (60.4) | 1632 (67.3) |  |
| No | 1703 (36.0) | 911 (39.6) | 792 (32.7) |  |

Abbreviations: NAPAIR, Nighttime/all-day physical activity intensity ratio; NAP, night-time active population; DAP, daytime active population; BMI, body mass index; FPIR, family poverty income ratio; OAPAI, overall average physical activity intensity

**Table S5.** Association of NAPAIR with all-cause and cardiovascular mortality in US adult

| **Mortality outcome** | **Death/No.** | **Hazard ratio (95% CI)** | | |
| --- | --- | --- | --- | --- |
|  |  | **Model 1^a^** | **Model 2^b^** | **Model 3^c^** |
| **All causes** |  |  |  |  |
| **NAPAIR** |  |  |  |  |
| ＜0.13 | 416/2300 | 1[Reference] | 1[Reference] | 1[Reference] |
| ≥0.13 | 404/2424 | 1.28 (1.15-1.47) | 1.35 (1.17-1.57) | 1.20 (1.02-1.40) |
| Per 0.1 increase | NA | 1.20 (1.12-1.29) | 1.18 (1.10-1.27) | 1.10 (1.01-1.20) |
| **Cardiovascular death** |  |  |  |  |
| **NAPAIR** |  |  |  |  |
| ＜0.13 | 86/2300 | 1[Reference] | 1[Reference] | 1[Reference] |
| ≥0.13 | 71/2424 | 1.01 (0.79-1.49) | 1.21 (0.87-1.69) | 1.00 (0.70-1.44) |
| Per 0.1 increase | NA | 1.11 (0.93-1.33) | 1.13 (0.95-1.34) | 1.03 (0.84-1.26) |

Abbreviations: NAPAIR, Nighttime/all-day physical activity intensity ratio;

^a^ Adjusted for age (as a continuous variable).

^b^ Additionally adjusted for sex, race/ethnicity**,** education attainment, marital status, family poverty income ratio (as a continuous variable).

^c^ Additionally adjusted for BMI (as a continuous variable), health status, smoking status, diabetes, hypercholesterolemia, hypercholesterolemia, and overall average physical activity intensity (as a continuous variable).
